# Combined micro X-ray absorption and fluorescence spectroscopy to map phases of complex systems: the case of sphalerite

**DOI:** 10.1038/s41598-019-55347-9

**Published:** 2019-12-11

**Authors:** Carlo Marini, Anna Maria Diaz Rovira, Nitya Ramanan, Wojciech Olszewski, Boby Joseph, Laura Simonelli

**Affiliations:** 1 CELLS-ALBA, Carrer de la llum 2-26, Cerdanyola del Valles, 08290 Barcelona, Spain; 2 Department de Fisíca i Departament de Química, UniversitatAutònoma de Barcelona, 08193 Cerdanyola del Vallés, Barcelona Spain; 3 Faculty of Physics, University of Bialystok, 1LK, Ciolkowskiego, 15-245 Bialystok, Poland; 4 Elettra-Sincrotrone Trieste, S.S. 14, Km 163.5 in Area Science Park, Basovizza, 34149 Trieste, Italy

**Keywords:** Materials chemistry, Materials science

## Abstract

Combining micro-X-ray absorption spectroscopy (μXAS) and micro-X-ray fluorescence spectroscopy (μXRF) is a promising approach for the investigation of complex multi-phase systems. In this work, we have employed this approach to investigate natural sphalerite, the most common form of Zinc Sulfide. Spatially resolved elemental distribution maps of common 3d metal atoms (Zn, Cu, Ni, Co, and Fe) are superimposed with chemical speciation and structural parameter maps in order to understand the sphaleriteore-formation process and metamorphosis. Chemical speciation and structural parameters have been obtained by analyzing the μXAS spectra collected in several representative points of the sample, after μXRF mapping. In the present case, this X-ray based approach has permitted to determine the spatial distribution of the Zn species in sphalerite. The presence of two main zincite and smithsonite inclusions has been established, with the latter located close to copper impurity center. Since copper is known to remarkably reduce the corrosion resistance of zinc, resulting in the formation of carbonate as the corrosion product, this implies a possible role of Cu in the growth of the carbonate inclusions. The results obtained highlight the efficiency of this method in univocally identifying the spatial distribution of phases in complex systems, thanks to the simultaneous access to complementary information.

## Introduction

The understanding of micron-scale processes and mechanisms in complex heterogeneous systems has naturally gathered a considerable effort in developing high resolution analytical probes to extract compositional, electronic, and structural information. In this regard, synchrotron-based X-ray absorption spectroscopy (XAS) and X-ray fluorescence spectroscopy (XRF) have become two of the most promising techniques for spatially-resolved studies, thanks to the advent of 3^rd^ generation brilliant sources and the improved focusing capability of modern x-ray optics, which easily permits beam sizes down to less than 100 μm. In contrast with other investigation methods requiring specific sample preparation, micro-XRF (μ-XRF) can be used to determine the elemental composition and distribution within the sample due to its relative simplicity and non-destructive character. Moreover, micro-XAS (μ-XAS) allows to distinguish between chemical species by accessing the local environment of the absorber in complex multiphase system. μ-XAS provides quantitative access to the local and electronic structures around a selected atom, such as its oxidation state, the number and nature of its neighbors, their distances, and the degree of order of the absorber’s local surroundings. Combining μ-XAS and μ-XRF is thus a desirable/necessary experimental approach in research problems, where the X-ray species’ selectivity and sensitivity are required for the non- destructive characterization of heterogeneous matrices.

Such a multi-technique approach can be applied to a variety of research fields. In this paper we demonstrate its potentialities, by presenting the results obtained on a sphalerite natural sample from the region of Santander (Spain). Sphalerite is a common cubic iron-zinc sulfide mineral (Zn,Fe)S, whose homogeneity range spans from 0 to ~56% of FeS in equilibrium with Fe metal^1^. Significant quantities of this mineral have been found in many parts of the world, including Australia, Myanmar, Germany, England, Italy, Spain, and Peru^2^. Due to its high zinc concentration, around 95% of all primary zinc used in metallurgy is extracted from sphalerite ores^3^. Sphalerite is found to incorporate a broad range of trace elements (Ag, As, Bi, Cd, Co, Cu, Fe, Ga, Ge, In, Mn, Mo, Ni, Pb, Hg, Sb, Se, Sn and Tl)^4^. In many ores, the substitution of Zn by these cations goes along with the presence of other additional mineral phases, likes galena, pyrite, calcite, dolomite, and fluorite. In particular, Cd forms both solid solutions and micro-inclusions inside minerals^5^, while Ag, Pb, Sb and Bi appear mostly as micro-inclusions of minerals carrying those elements. Elements like As Se, Au, and Ge are found in minor amounts as few hundreds of ppm. Other elements like In and Sn are generally coexist in sphalerite and their presence is found to correlate with Cu and Ag contents^4^. In case of 3d metals, sphalerite usually accommodates high concentration of Mn, Fe, Co, Ni, and Cu, mainly by simple cations exchange (Zn^2+^→ M^2+^)^4,6^. Such rich elemental composition leads to the formation of discrete domains whose sizes range in scale from centimeters to micrometers^7^.

Generally, the visual inspection of sphalerite reveals many colored bands^8,9^. Because of the geological implications, the compositional ores zoning of sphalerite has been extensively studied in the last decades^10,11^. It has been claimed that the internal zoning reflects non-equilibrium self-organization processes that could occur during the mineral growth^12^. Moreover, it has been demonstrated that in sphalerite, phase coexistence leads to variation in crystal lattice size and structural disorder of the constituting phases. In particular, Barton and Toulmin^13^ established a relation between the lattice parameter of the sphalerite and temperature and pressure conditions of its formation. The applicability of this method and its restrictions can be found in ref. ^14^ and references therein. More recently Pring *et al*. have shown that substitution of Zn by Fe creates distortion of the sphalerite structure as well as changes in the cell parameters^15^. The determination of the local structural properties and the spatial distribution of coexisting species in the mineral are thus essential to shed light on the ore-formation process and metamorphosis. Here we address this issue, combining μ-XRF elemental mapping and μ-XAS at the Zn K-edge. We have unveiled the spatial distribution of Zn-phases by simultaneous linear combination fit (LCF) of the X-ray Absorption Near Edge Structure (XANES) spectra and first shells modeling of Extended X-ray Absorption Fine Structure (EXAFS) data. The superimposition of the μ-XRF, the LCF and the EXAFS parameter maps permits the determination of the correlation between first neighbor distances, structural disorder and chemical composition.

## Materials and Methods

For this investigation a natural sphalerite mineral has been collected from Santander area of Spain, cut into approximately 8 × 4 × 2 mm^3^ plate. A full characterization of the sphalerite source ore from Cantabria in Spain can be found in ref. ^16^. XAS and XRF spectra were recorded at the CLÆSS X-ray absorption and emission spectroscopies beamline at Alba (Barcelona, Spain)^17^. The synchrotron radiation emitted by a multipole wiggler has been vertically collimated by a mirror and monochromatized by a liquid nitrogen cooled double crystal Si(111) monochromator. The beam size at the sample position was ~100 × 100 μm^2^, thanks to the toroidal mirror focusing and the partial beam cut of the sample slits. Higher harmonics contribution to the desired energy has been eliminated by properly selecting the coating and the rejection angle of the two mirrors. The beamline has been calibrated by measuring a Zn foil. The measurements of standards have been realized in transmission mode by counting incident and out-going photon fluxes with respect to the sample using gas-filled ionization chambers. μXAS spectra and μXRF elemental maps have been recorded by selecting the Kα fluorescence lines of Zn, Cu, Ni, Co, and Fe in the XRF spectrum of the 5 elements Silicon Drift detector (from Quantum Detector). Fluorescence and emission spectra have been collected in 45 degrees (with respect to the incoming photon beam) geometry. For these maps a 10.5 keV incoming beam has been used with 100-ms counting time per point. μXRF maps were collected in a grid of 100 × 100 μm^2^.

XAS data have been recorded along the sample and processed according to standard procedure. For each spectrum, the raw data has been normalized by subtracting and dividing pre-edge and post-edge backgrounds as low order polynomial smooth curves. Linear combination fits of the obtained XANES was then performed in the range 9655 eV to 9675 eV. The normalized sum of square residuals was used as a measure of the discrepancy between the unknown data and the fit model. The EXAFS signal has been extracted and modeled by the standard theory equations. A detailed review about data modelling and its application to geophysics can be found in ref. ^18^.

The useful k-range available for the EXAFS analysis was between 2.8 Å^−1^ and 8.5 Å^−1^. A sine window has been used to truncate the EXAFS signal. Fourier transformed (FT) signal from the k^2^-weighted EXAFS data was used for visualization of the real space distribution of Zn local structure.

Due to the large number of spectra to deal with, a python interface to the IFEFFIT program^19^ has been written in order to batch process the raw data directly from reading the file, XAS normalization, LCF of the XANES, EXAFS signal extraction, and finally Fourier transforming and fitting.

## Results and Discussion

**Figure 1:**
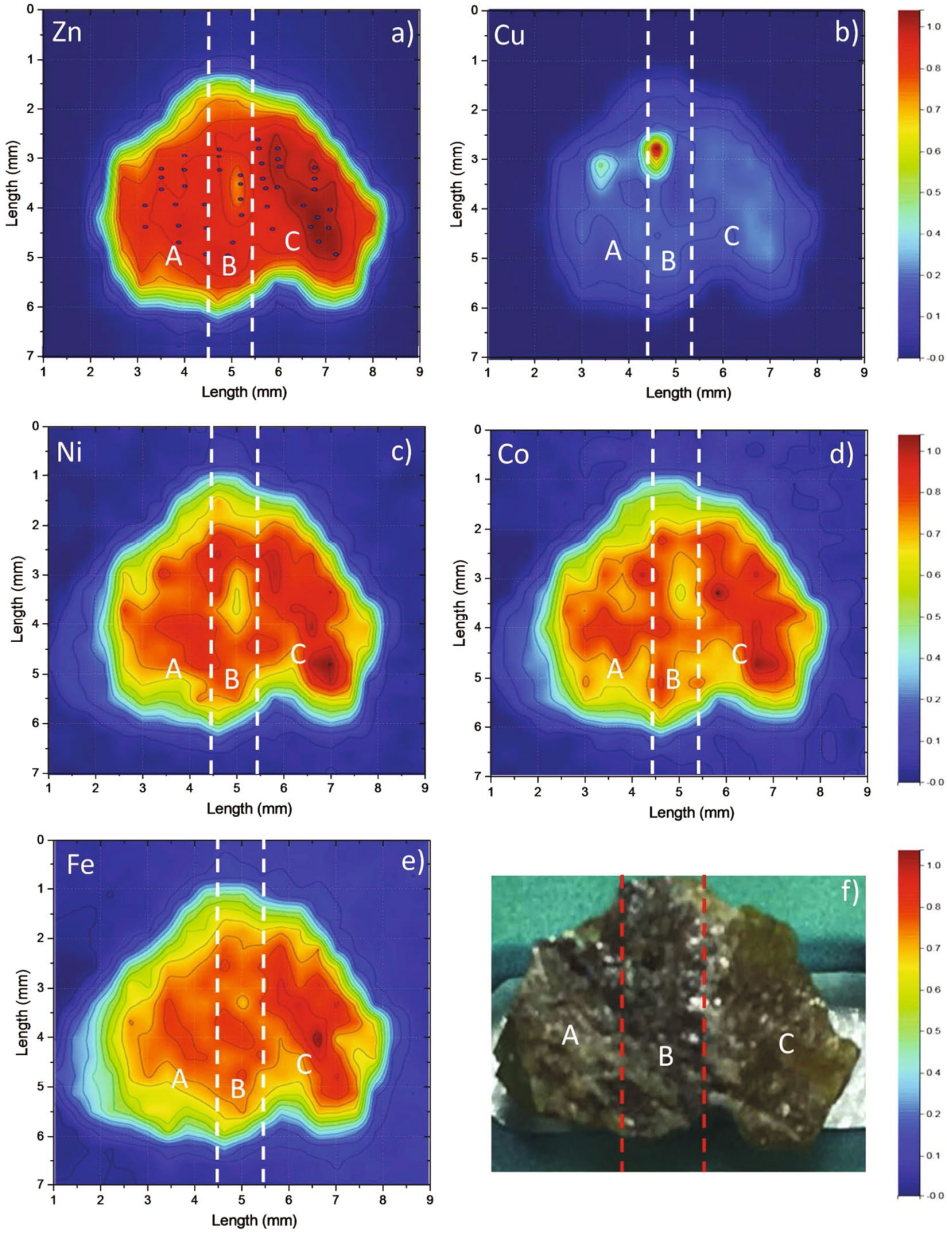
Zn, Cu, Ni, Co, Fe X-ray florescence maps (**a**–**e**) of the sample. Scanning step: 100 × 100 μm^2^; count time: 100 ms/point. The points in Zn map represent position where EXAFS spectra have been acquired. The elemental maps intensity is normalized to be in between 1 and 0 for a better comparison. (**f**) A photograph of the mineral indicating the regions A, B and C.

Elemental maps of the sample are shown in Fig. 1 for Zn, Cu, Ni, Co and Fe, together with a picture of the sample. Except for the Cu case, for which two impurity centers are clearly detectable, all the other transition metals show similar elemental distribution. In particular, the Fe distribution seems to match the one of Zn along the entire mineral^1^, with an average value of the ratio Fe counts vs Zn counts of 0.022 ± 0.005. The common elemental distribution of all the transition metals investigated could suggest more complicated stoichiometry of the solid solution (Fe,Zn)S, characterized by the presence of other 3d metal complexes. To easily describe the obtained results, we group the data in three regions labeled as A, B and C (note the two super-imposed vertical lines passing at 4.5 mm, 5.5 mm in Fig. 1f), according to the zone color distribution along the sample. The A region on the left side appears visually as dark green in color. In its top part, a Cu impurity center is present, combined with a minimum content of the other transition metals. For the rest of the A region all the elemental distributions appear to be almost uniform. The B region is black in color with few white spots and appears to be the darkest one in the entire mineral. In the top part of B, another Cu impurity is identifiable. Similar to the A region, close to the Cu impurity we can identify a minimum in concentration for all the other investigated elements. Finally the C region is the lightest in color amongst the three regions. It appears to be the richest one in Zn, Ni, Co, Fe, and Cu, if we do not consider the two Cu impurity centers. The bottom part of C is characterized by a relatively higher transition metal concentration. These findings can be explained considering that, differently from the other 3d metals, only Cu does not usually incorporated into the ZnS structure^14,20^, unless as part of a coupled substitution and/or excretions mineral. In these later case, chalcopyrite (CuFeS_2_) may be formed as a result of the replacement of sphalerite as well as by co- precipitation of both mineral^21^.

**Figure 2:**
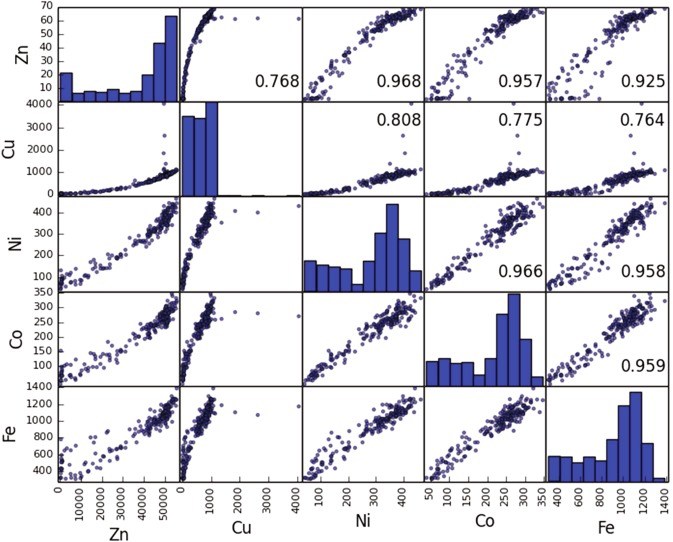
Zn, Cu, Ni, Co, Fe X-ray fluorescence scatter matrix. Values of the ρ are also reported.

In order to characterize quantitatively the metal distributions in the sample and to elucidate the existing correlation between them, we built the scatter matrix^22^ and calculated the Pearson correlation coefficient ρ^23^ between the detector counts measuring the fluorescence lines corresponding to the different metals. A ρ value of 1 or −1 implies that a linear equation describes the relationship between the counts belonging to *two* fluorescence line. ρ = 0 implies that there is no linear correlation between the variables. Figure 2 reports the obtained scatter matrix calculated taking into account the whole mineral area. It shows a strong inter-dependence between all the metals except for copper, because of the two isolated impurity centers, in agreement with the visual observation of the elemental distribution maps. Remarkably, all the elemental distributions (shown on the diagonal of the scatter matrix) are bimodal again with the exception of copper. These findings are also confirmed by the Pearson correlation coefficient values, reported in Table 1. We notice that in all the cases, except for Cu, all the elements are highly positively correlated (ρ > 0.92). In these cases, a closer inspection reveals the Fe-Zn correlation to be the worst, while the Ni-Co, Ni-Fe, and Fe-Ni, show the best linearity despite a bit of scattering along it. Moreover in the Zn-Fe, Zn-Ni and Zn-Co correlations it is possible to identify two concentration regions corresponding to different linear slopes and to the two regions of the bimodal elemental distribution. Most likely the two different regions (high and low transition metal content) correspond to the presence of different phases or different phase-ratios. Repeating the scatter matrix calculation in the different regions of the mineral provides similar results, enlightening that correlations register their maximum value in the C region and its minimum in the B region.

**Table 1:** Pearson correlation coefficient between fluorescence counts.

	Zn	Cu	Ni	Co	Fe
Zn	1.000	0.893	0.991	0.987	0.976
Cu		1.000	0.903	0.888	0.874
Ni			1.000	0.991	0.987
Co				1.000	0.989
Fe					1.000

**Figure 3:**
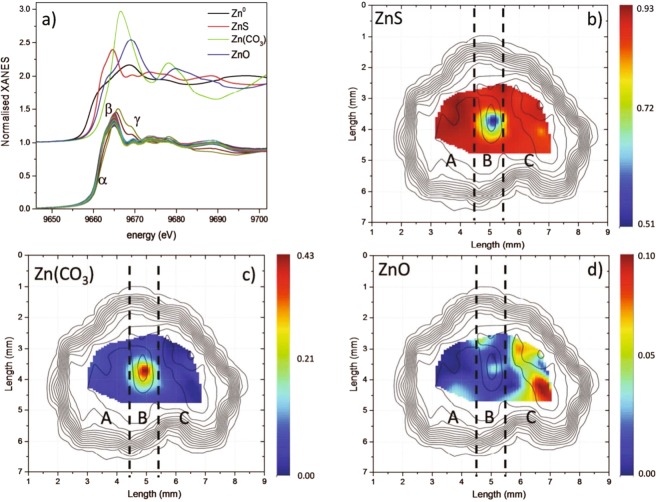
(**a**) Zn K edge XANES spectra collected on the sphalerite sample, (**b**) ZnS, (**c)** ZnCO_3_ (**d**) ZnO fractions spatial distribution as obtained from LCF of standards superimposed to Zn elemental distribution contour plot.

Zn K-edge μXAS spectra were acquired from selected spots within the sample area (indicated as dots in Fig. 1a). Due to the high concentration of Zn in the sample, the data have been corrected for self-absorption effect (responsible for dampening and distortion of the spectral features)^24^. Subsequently for the results reported below, self-absorption has been estimated by considering a simplified stoichiometry for the mineral (ZnS) and the data have been processed by the *fluo* code^25^. Corrected normalized XANES spectra are shown in Fig. 3a, together with several standards: Zn, ZnS, ZnCO_3_, and ZnO. It is possible to describe the Zn K-edge XANES spectra of sphalerite by focusing on the evolution of three main features, labeled as α, β, and γ which are located at 9662.6, 9665.2, and 9669.5 eV, respectively. By comparing the sample with the references spectra, it appears that features α and β correspond mainly to ZnS, while γ to ZnO. Moving along the sample leads to changes in the relative intensities of these features and at the same time their energy position, especially for β, which moves towards higher energy values in some spectra.

These findings correspond to an inhomogeneous distribution of Zn species in natural sphalerite mineral. In order to provide a quantitative estimate, the spectra were analyzed by linear combination fit (LCF) of the proposed standard compounds. For each reference compound we built a map showing the corresponding relative fraction as a function of the position in which the spectra are collected. Figures 3b–d report the spatial distribution of the reference compounds fractions, superimposed with the contour plot of the Zn elemental map.

As expected the analyzed sphalerite sample is mainly composed of ZnS (minimum fraction: 51%, maximum fraction: 93%). ZnO (<10%) is mainly concentrated in the C region, while ZnCO_3_ is found mainly as an impurity in the center of the sample (B region). Interestingly the ZnCO_3_ inclusion is found to grow up close to the Cu impurity center. This suggests a role of the copper cluster in the growth of the carbonate inclusions, since the presence of copper remarkably reduces the corrosion resistance of zinc^26^, with carbonate is the most probable corrosion product in the presence of CO_2_^27^. ZnCO_3_ and ZnO are, in fact, two common minerals that are found in association with sphalerite. ZnCO_3_ can appear due to carbothermic reduction of sphalerite in the presence of sodium or calcium carbonate^28^, while ZnO has been found to crystallize as an additional phase from ZnS in the presence of oxygen as the temperature is raised up 500 °C^29^. Finally we notice that the fraction of ZnO is higher where the concentration of the transition metals reaches its maximum (see comparison between Fig. 1a–e,d).

**Figure 4:**
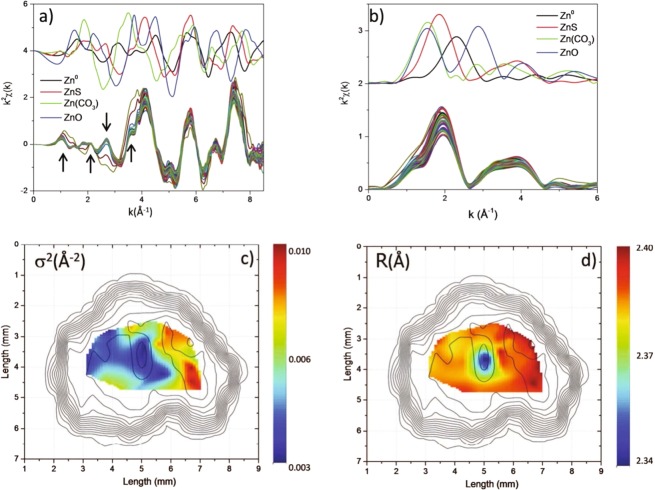
(**a**) Zn K edge EXAFS spectra and (**b**) relative FT collected on the sphalerite sample together with reference compounds’ signals. Spatial distribution of Debye Waller factor (**c**) and distance (**d**) superimposed to Zn elemental distribution contour plot.

Figures 4a,b shows Zn K-edge EXAFS spectra and their corresponding Fourier transforms (FT), together with the signal obtained for the four reference compounds. Despite the small k-range (Δk = 5.7 Å^−1^), two main structures, located around 1.9 Å and 3.6 Å and corresponding to first and second shells can be identified in the FT signal, similar to ZnS (red curve in Fig. 4b).

In addition to the ZnS contribution, a second type of EXAFS pattern can be identified. Some of the spectra present distinct features at very low k (i.e. in the XANES region) as well as in between k = 3 and k = 4 Å^−1^ (see the arrows), which, interestingly, match those of ZnCO_3_ (green curve of Fig. 4a). This is reflected in the FT signal in which, apart from the intensity, we notice that the first FT peak (located around R = 2 Å) shifts towards lower values for few spectra. This is clearly related to the presence of species showing shorter first neighbor distances, such as ZnO and ZnCO_3_.

In order to get quantitative information from the EXAFS spectra, we have performed a first shell fit. Since the major component of the XAS spectrum is zinc sulfide, a zinc sulfide cluster has been considered for the first shell EXAFS data analysis. Results of the fitting procedure are shown in Fig. 4c,d for Debye Waller factor (σ^2^) and first neighbor distance (R), respectively. Also, in this case three different regions can be identified. The local structure of Zn looks slightly more contracted in the left part of the sample (region A). A clear minimum of R appears in the central region (B regions). Bigger R values are detected in the right part of the sample (C region). σ^2^ is directly related to the structural disorder and the number of the atoms surrounding the absorber. It turns out that the shrinking of the first neighbor distance is associated with a decrease of σ^2^.

Let us now compare the results obtained by looking at the XANES and EXAFS regions and the detected elemental correlations. We observe that the minimum R and σ^2^ are found in the region of the sample where the fraction of Zn(CO_3_) appears to be maximum, as shown by XANES LCF in Fig. 3d.

For the central region of the sample (B), the results of the EXAFS and XANES analysis are coherent and can be easily explained. Zn(CO_3_) has a contracted first shell with respect to the majority fraction of ZnS, which results in the reduction of average first neighbor distance (see panel Fig. 4b where the shift of the first peak in FT is evident). Moreover, the decrease of σ^2^ in the central region of the mineral can be interpreted as an increase in the coordination number of first neighboring atoms, since the structure is changing from tetrahedral (ZnS) to octahedral (ZnCO_3_). On the other hand, the maximum R and σ^2^ are found in region C where the ZnO reaches its maximum fraction (Fig. 3c). In this zone with high content of transition metals, all the elemental distributions appear to be strongly correlated. In region C, the maximum coexistence between ZnS and ZnO species is derived from the XANES LCF. This can be considered contradictory, since the presence of a ZnS-ZnO mixture is expected to result in the contraction of the first shell distance, since Zn-O bonds are shorter than Zn-S ones (see Fig. 4b). However, in contrast with Zn(CO_3_) (where the fraction is around 43%) the fraction of ZnO is quite low (<10%), which means that the oxide can be considered as an impurity inside the ZnS matrix. This is coherent with the low miscibility of the two compounds^30^. This makes the situation more complex, since strain effects due to coexistence of the two minerals intervene^31^. Moreover, the cluster size of ZnO–ZnS solid solution depends on dimensionality and growth conditions^32–34^. Finally, since Ni and Co increase their percentages in this area of the sample, other metal complexes can also act as impurities on the ZnS structure (see comparison between Figs. 1c,d, 4c,d). All these arguments can be invoked to be responsible for the expansion of the Zn-ligand distance. In coherence with this scenario, the structural disorder (i.e. σ^2^) increases.

## Conclusions

In this study, we present a combination of µXRF and µXAS data analysis techniques as an effective method to unveil the speciation distribution in heterogeneous structures. We have exploited the study of Zn speciation in natural sphalerite mineral to show the powerfulness of the approach. By means of µXRF, Zn and other metal species (Cu, Ni, Co, and Fe) have been identified and spatially distinguished in the sample matrix. With the exception of Cu, for the all metal species investigated we have observed a Pearson correlation coefficient >0.92, indicating a strong link between the elemental distributions of the 3d metal inside the analyzed sample.

µXANES analysis has been performed in terms of linear combination fits of Zn standards. Apart from zinc sulfide (which represents the highest fraction between the Zn species, being always above 50%), zincite (ZnO) and smithsonite (ZnCO_3_) have been identified in specific regions of the natural sphalerite. µEXAFS has been then employed in order to structurally characterize the Zn local environment in terms of the nearest-neighbor distances and bond order and to confirm the results. Remarkably the presence of zincite and smithsonite inclusions affects the Zn local geometry differently: while smithsonite induces a shrinking of distances and increase of order, zincite leads to the opposite behavior. Thermodynamic implications in terms of mineral growth conditions are discussed.
